# Biological Detoxification of Mycotoxins: Current Status and Future Advances

**DOI:** 10.3390/ijms23031064

**Published:** 2022-01-19

**Authors:** Lu Liu, Mei Xie, Dong Wei

**Affiliations:** 1 Guangdong Provincial Key Laboratory of Microbial Culture Collection and Application, State Key Laboratory of Applied Microbiology Southern China, Guangdong Institute of Microbiology, Guangdong Academy of Sciences, Guangzhou 510070, China; luckyliulululu@163.com; 2 Engineering Research Center of Starch and Vegetable Protein Processing Ministry of Education, Guangdong Province Key Laboratory for Green Processing of Natural Products and Product Safety, School of Food Science and Engineering, South China University of Technology, Wushan Road 381, Guangzhou 510641, China; 3 Food Science and Technology Program, Beijing Normal University-Hong Kong Baptist University United International College, Zhuhai 519087, China; xmei1202@163.com

**Keywords:** mycotoxins, biodegradation, probiotics, recombinant enzyme, microbial consortia

## Abstract

Mycotoxins are highly toxic metabolites produced by fungi that pose a huge threat to human and animal health. Contamination of food and feed with mycotoxins is a worldwide issue, which leads to huge financial losses, annually. Decades of research have developed various approaches to degrade mycotoxins, among which the biological methods have been proved to have great potential and advantages. This review provides an overview on the important advances in the biological removal of mycotoxins over the last decade. Here, we provided further insight into the chemical structures and the toxicity of the main mycotoxins. The innovative strategies including mycotoxin degradation by novel probiotics are summarized in an in-depth discussion on potentialities and limitations. We prospected the promising future for the development of multifunctional approaches using recombinant enzymes and microbial consortia for the simultaneous removal of multiple mycotoxins.

## 1. Introduction

The continuous occurrence of food contaminants worldwide poses a critical threat to the health of human and livestock. One of the major contaminants in food and feed products are mycotoxins, the secondary metabolites synthesized by toxigenic fungi strains, mainly those belonging to *Penicillium*, *Aspergillus*, *Alternaria* and *Fusarium* genera. Both acute and chronic exposure to mycotoxin-contaminated food may cause deleterious health effects including retarded growth, suppression of the immune response, vomiting, infertility and gastrointestinal and carcinogenic diseases [1]. These mycotoxins occur in various products, from raw agricultural products such as corn, barley, oats, fruits and herbs, to commercial commodities including aquafeeds, beverages, fruit and vegetable-derived products [2,3,4]. The contamination of mycotoxins can occur during any part of the complex food chain, including harvest, industry processing, transportation and/or storage, imposing social burdens on the food industry due to the waste created by contaminated products [5]. This creates an urgent demand for mycotoxin removal methods to minimize economic loss and hazards to consumers.

**Figure 1:**
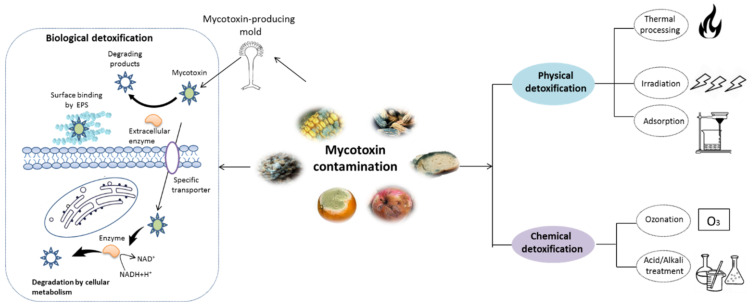
Schematic representation of mycotoxin detoxification methods. The physical methods mainly include thermal process, irradiation, and adsorption techniques, while the chemical methods involve in the treatment with acid/alkali solution and ozonation. The major mechanism of biological detoxification involves in the surface binding by extracellular polymeric substances (EPS), degradation by enzyme and cellular metabolism.

As shown in Figure 1, various strategies have been reported for mycotoxin removal, which can be roughly categorized into physical [6], chemical [7], and biological methods [8]. Although some physical and chemical approaches can remove mycotoxin with various degree of success, some problems, such as potential safety issues, loss of important nutrients and high costs, still hamper their application in the food industry [9]. In recent years, various biological detoxification approaches, particularly those using microbial cells or enzymes, have been demonstrated to be highly effective in degrading mycotoxins into less toxic products with high specificity [1,10]. Compared with the physical and chemical methods, the detoxification processes by microbes or enzyme can be handled under mild working conditions with fewer losses to the nutritional quality of food products [11,12]. To date, various microorganisms including bacteria and yeast have been found to be capable of degrading mycotoxins [13,14,15,16]. Additionally, mycotoxin-degrading enzymes obtained by direct extraction and purification from biomaterials or expression in mature microbial hosts by genetic engineering technique have been well documented [17,18]. Nonetheless, a limited number of studies have been developed to evaluate the potential safety derivatives or metabolites during the detoxification process, although the byproducts can be more harmful than the initial product [19]. Moreover, the mechanisms involved in mycotoxins detoxifications are not straightforward. Therefore, it is essential to address these questions before the real applications of biological agents.

The aim of the current review is to address some of the developments on (a). Novel mycotoxin-removal approaches, notably based on the promising probiotics, recombinant enzyme and microbial consortia, and the main mechanisms of action underlying the degradation process; (b). New strategies for the elucidation of the degradation pathway and a risk assessments of the degrading by-products; (c). Challenges and limitations in the application of biological mycotoxin removal.

## 2. Chemical Structure and Toxic Effects of Mycotoxins

Since 1960, more than 400 forms of mycotoxins have been identified and reported. The most common mycotoxins in food include aflatoxins (AFs), trichothecenes, ochra-toxins, zearalenone (ZEA), fumonisins, and patulin, which are highly toxic [20]. These notable contaminants can be produced by various toxigenic fungi due to improper harvesting, storage or transport conditions [21]. Most of them have complex chemical structures containing specific groups that confer the toxic effects. Basic information of the main mycotoxins including chemical structure, biological effect and producing fungi is summarized in Table 1 [21,22,23,24,25].

### 2.1. Aflatoxins (AFs)

Aflatoxin (AFs) is the most widely studied mycotoxin encountered in agricultural commodities. They are mainly produced by *Aspergillus* species, *Aspergillus flavus*, *A. parasiticus* and *A. nomius*. AFs shave been reported to induce hepatic toxicity, nephron toxicity and immune suppression in humans [26]. Of the AFs, AFB1 is extremely hazardous and has been considered as a Group I of naturally occurring carcinogens [27]. AFs are furanocoumarins associated with a bisdihydrodifuran or tetrahydrobisfuran united to a coumarin, replaced by a cyclopentanone or a lactone [28]. Lactone ring and the double bond in the terminal furan ring of AFs are responsible for the toxicity and carcinogenic activity [23,29].

### 2.2. Trichothecenes

As one of the major classes of mycotoxins, trichothecenes (TCNs) cause a significant economic impact on the food chain each year. TCNs are formed in nature by some filamentous fungi such as *Aspergillus*, *Penicillium*, *Fusarium*, *Spicellum* and *Stachybotrys*, among them *Fusarium* is the most common toxin source in cereals, vegetables, and feedstuff [30,31]. The consumption of TCNs-contaminated food may affect the gastrointestinal tract, kidney, liver, and immune system, and cause health issues such as anorexia, vomiting, abdominal pains and cardiovascular dysfunction [32]. TCNs are tetracyclic sesquiterpenoid epoxides and form a family of over 200 toxins with an atricyclic 12,13-epoxytrichothec-9ene (EPT) core structure in common [33]. According to their structure, toxins in this family are classified into four groups (types A, B, C, and D) based on the substitution pattern of EPT, among them, type A and type B are of special interest because of their high toxicity [34]. Representative type ATCNs includes T-2 toxin (T-2), HT-2 toxin (HT-2) and neosolaniol (NEO), while type BTCNs were represented by deoxynivalenol (DON) and its acetyl derivatives [35]. The 12, 13-epoxide ring, 9–10 double bond and acylated side groups in trichothecenes are essential for imparting the toxic effects in inhibiting the synthesis of protein, RNA and DNA [36,37]. Generally, the complete detoxification of TCNs is a challenge due to their stable and complex structure [38].

### 2.3. Ochratoxins

Ochratoxins (OTs) belong to a family of mycotoxins commonly found in various food and feed mainly produced by *Penicillium* and *Aspergillus* species, including *P. verrucosum*, *P. nordicum*, *A. ochraceus*, *A. carbonarius* and *A. niger* [39]. They comprise a group of coumarin derivatives, including ochratoxin A (OTA), ochratoxin B (OTB) and ochratoxin C (OTC), of which OTA is the most prevalent and toxic compound [40]. OTA is of neurotoxicity, carcinogenesis, immunotoxicity and hepatotoxicity, which poses a serious threatens to both human and animal health. [41]. Isocoumarin moiety is the key toxic group of OTA, and the carboxyl group of the phenylalanine moiety as well as the Cl group contribute to its toxicity [25].

### 2.4. Zearalenone

Zearalenone (ZEA) is a kind of nonsteroidal mycotoxin that contaminates a variety of cereals [42]. ZEA is a secondary metabolite that is synthesized by several species of mold fungi belonging to the *Fusarium* family that colonizes plants grown in the temperate climate zone such as corn, maize and other grain crops [43]. ZEA belongs to xenoestrogens that have a chemical structure similar to natural estrogens. Therefore, tZEA can bind to estrogen receptor sites, causing hormonal imbalance that may lead to reproductive diseasesin human and livestocks [44]. Lactone group in the chemical structure of ZEA is critical to the toxicity of ZEA. It has been reported that the toxic effects of ZEA can be significantly reduced by the cleavage of the lactone ring [45].

### 2.5. Fumonisins

Fumonisins (FBs), polyketide-derived mycotoxins, are mainly produced by *Fusarium* mold species that commonly infect corn and other agricultural products [46]. Based on the structural difference, four main groups of fumonisin analogs have been characterized, and among them, FB1 is the most prevalent and toxic [47]. FBs have a chemical structure similar to sphingolipid long-chain bases, such as sphinganine (Sa) and sphingosine (So) which are intermediate compounds in the sphingolipid metabolism, which can inhibit ceramide synthase and block the biosynthesis of complex sphingolipids, resulting in a wide spectrum of pathological effects in humans and animals [48]. The tricarballylic acid (TCA) side groups and the free amino group play important roles in FBs toxicity mechanism. It has been found that the removal of these groups can significantly reduce both phytotoxicity and mammalian cytotoxicity [49,50].

### 2.6. Patulin

Patulin (PAT) is a tetraketide mycotoxin most commonly found in apples and apple-derived products [51]. It is produced by a variety of filamentous fungi including *Penicillium*, *Aspergillus* and *Byssochlamys* [52]. Acute PAT exposure damages gut epithelium, liver, kidney, and immune system, causing symptoms such as nausea, vomiting and intestinal hemorrhages [53]. PAT has also been shown to affect the activities of thiol-containing enzymes that play important roles in glycolysis, since this mycotoxin can easily form covalent sulfhydryl-containing compounds, which is the main mechanism associated with PAT toxicity [54]. The destruction of lactone, furan or the pyran ring and hemiacetal in the structure of PAT may be an indicator of toxicity reduction [8].

## 3. Mycotoxin Removal by Probiotics

### 3.1. Fast Glance to Probiotic Properties

Probiotics, defined as ‘‘live microorganisms which when administered in adequate amounts confer a health benefit on the host’’ by the World Health Organization (WHO), have been incorporated into various kinds of products such as foods, drugs, and dietary supplements [55]. Beyond their basic nutritional value, probiotics provide numerous benefits such as the alleviation of lactose intolerance, maintaining balance of the digestive tract and modulation of inflammatory responses [56]. The most extensively studied and widely used probiotics include *Lactobacillus*, *Bifidobacterium*, yeast *Saccharomyces cerevisiae* as well as some *Bacillus* species [57]. Gaggìa et al. (2010) summarized the species of probiotics and their health-promoting characteristics based on extensive studies and an internet search of commercial products [58]. These nonpathogenic, nontoxigenic, and fermentative microorganisms are quite commonly added to “functional foods” and growth supplements for human health. The application of probiotics is also prevalent in the aquaculture field, acting as alternatives for antibiotics or disinfectants to improve water quality [59]. Moreover, specific strains of probiotic bacteria were indicated to be effective in removing contaminants such as heavy metals, pesticides as well as mycotoxins, which might act as promising bio-agents for food safety [60,61,62].

### 3.2. Mycotoxins Detoxification by Probiotics and the Related Mechanism

The biological detoxification of mycotoxins by probiotic bacteria was reviewed years ago [28,55,59]. Probiotics, particularly lactic acid bacteria (LAB) and yeasts such as *Saccharomyces* genus, can remove mycotoxins primarily through two mechanisms, surface adsorption or biodegradation (Figure 1). Toxins removal by surface adsorption of microorganisms is a fast and reversible process that does not cause any chemical changes in the substrate. In contrast, biodegradation is permanent but results in undesirable metabolites.

#### 3.2.1. Cell-Binding of Mycotoxins

In the last three decades, numerous studies have been developed regarding toxin binding by the cell-surface of probiotics such as *Lactobacillus rhamnosus*, *Lactobacillus amylovorus*, *Lactobacillus plantarum*, *Lactobacillus pentosus* [63,64,65,66]. Most of studies in vitro have been carried out using viable cells or heat-treated cells with aqueous toxic standards. The binding capacity of mycotoxins was strain-dependent, and depends on the natural structure of the toxin as well as physicochemical conditions. In vivo studies in animal trials and human clinical trials investigating the real effect of probiotics binding capacity as antidote for mycotoxin detoxification have been reviewed [67]. Although certain probiotic strains have shown a positive role in the restoration of mycotoxins damage, studies concerning the fate of the ingested mycotoxins and probiotic-mycotoxin still need to be supplemented.

The structural components of the probiotic cell wall play a pivotal role in the binding of toxins by microorganisms. LAB has the typical cell wall structure of Gram-positive bacteria, which consists of thick and multilayered peptidoglycan (PG) sacculi decorated with other glycopolymers including teichoic acids (TAs), polysaccharides (PSs) and proteinatious Slayer [68]. The binding efficiency of different LAB species seems most closely related to the amino acid sequence of peptide bridges of the PG [69]. The cell wall of LAB contains plenty of negatively charged functional groups that might facilitate the binding capacity due to the presence of S-layer proteins [60]. The literature available has limited information on the role of teichoic acids and polysaccharide involved in the toxins binding mechanism, experimental studies of enzymatic degradation showed that deficiency of these components in cell wall leads to less toxin accumulation [70,71]. The cell wall of yeasts such as *Saccharomyces* genus is mainly composed of an inner layer with β-glucans and chitin, and outer layer with heavily glycosylated mannoproteins. Some studies suggest that the cell wall thickness depends on β-D-glucans reticular organization and that β-(1, 3)-D-glucans contents play a major role in the toxin adsorption efficacy in *S. cerevisiae* [72,73]. Thus, elucidating the differences between cell wall components of probiotics species might provide a potential strategy to select strains to act as the mycotoxin binder.

#### 3.2.2. Biodegradation

In contrast to cell-binding methods, research on the biodegradation of mycotoxins by probiotics is very limited. The available literatures about mycotoxins degradation by probiotics over the last decade was summarized in Table 2. Harkai et al. (2016) successfully selected several *Streptomyces* strains which have shown a strong capability to degrade AFB1 and ZON mycotoxin with eliminated genotoxicity [74]. Rao et al. (2017) reported a well-known probiotic bacteria *Bacillus licheniformis* isolate CFR1, which can degrade aflatoxin B1 in liquid culture media with a reduction rate of 94.7%. In the same study, it was found that AFB1 could be metabolized to degradation products using a cell-free supernatant, and that treatments such as heating, proteinase K and SDS lead to the complete loss of degradation activity, indicating that the extracellular proteins or enzymes may be involved in the degradation process [75]. The application of biological detoxification in food fermentation has enormous practical significance. Juodeikiene et al. (2018) treated wheat grains with three P. pen-tosaceus strains, and the results showed a decrease in mycotoxins (DON, T-2 and HT-2 toxins) in malting wheat grains. The strains would serve as candidates for reducing mycotoxins in corresponding raw materials for beverages and certain baked-goods production. [76]. During the beer-brewing process, some stable mycotoxins in contaminated cereal-derived raw materials may survive and enter the final products, thereby it is important to screen brewers’ yeast with the ability to alleviate toxicity. In the study conducted by Nathanail et al. (2016), the lager yeast *Saccharomyces pastorianus* A15 may enzymatically transform *fusarium* trichothecenes to less toxic form in the fermentations of brewer’s wort, with reduction rates of 15% for DON, 17% for DON3Glc, 34% for HT-2 and 31% for T2 [77]. This strain might be an excellent candidate for mycotoxicosis control during food processing. It must be addressed that the biodegradation method may bring potential risks. Most of the studies did not look into the possible toxicity of by-products created during the decomposition process. For biotechnological utilization, applicable methods must be developed to monitor the potential hazardous metabolites and biological effects in mycotoxin biodegradation.

## 4. Biodegradation of Mycotoxins by Microbial Consortia

The biodegradation of mycotoxins that are able to transform mycotoxins into nontoxic metabolites has emerged as an alternative strategy for food and feed-safety control. However, it is difficult or even impossible to completely detoxify some complex contaminants such as aromatic compounds and mycotoxins by single-species strains. Therefore, toxin biodegradation with microbial consortia has gained increasing attentions, owing to their ability to perform more complex tasks than individual strains or species [78]. Moreover, microbial consortium is more resilient to environmental changes than single microbial species. For instance, constructed PAHs degrading microbial consortia has a higher potential for bioremediation of sites pollution due to their elevated tolerance and improved adaptation [79]. Microbial consortia have become a major strategy for soil-pollutant degradation; however, the degradation mechanism remains largely unknown. Available literatures showed that the application of microbial consortium might provide a new solution for mycotoxin biodegradation, which is a complex process involving two or more tasks that in some cases may pose insuperable challenges for one single organism.

A diversity of microbes in soil with great diversity has been reported to bio-transform complex contaminants into harmless or less toxic smaller molecules. The possible role of soil microbial communities for mycotoxins degradation was suggested by several researchers. In the early research of Beeton and Bull (1989), soil bacterial communities was found to be able to utilize T-2 toxin as a sole carbon source and energy, and the major degradation pathway involved HT-2 toxin and T-2 triol production by side chain cleavage of acetyl moieties. Compared to single bacterial function, the complete communities were more active in terms of their T-2 toxin degradation rate [80]. In their study, they isolated a bacterial consortium PGC-3 from soil by in situ enrichment and found that PGC-3 exhibited strong de-epoxidation activity on trichothecene mycotoxins under aerobic conditions. The result of the 16S rDNA sequences analysis showed that PGC-3 comprised 10 bacterial genera, among them, *Desulfitobacterium* increased steadily under increased DON concentrations [81].

Besides, thermophilic agricultural compost materials with abundant microorganisms were also used as the microbial source. In the work of Wang et al. (2020), a novel microbial consortium (NZDC-6) from agricultural composts with moldy corncob and cornstalk was characterized to degrade estrogenic ZEA and its cognates, α-zearalenol (α-ZAL) and β-zearalenol. This consortium was found to be thermophilic and highly effective with a degradation ratio >90% at an optimum temperature of 60 °C. The author further evaluated the potential effects of NZDC-6 on the treatment of ZEA-contaminated corncobs by semisolid fermentation. Fermentation conditions have a great influence on microbial composition and efficiency. It was found that a high solid/liquid ratio lead to the increase in dissolved oxygen content and acidity, thus inhibiting microbial ZEA degradation [82]. Zhao et al. (2019) isolated a FB1-degradingbacterial consortium SAAS79, mainly consisted of *Pseudomonas*, *Comamonas*, *Delftia*, *Sphingobacterium*, and *Achromobacter* genera, from spent mushroom compost. SAAS79 could transform FB1 to less toxic degradation products in 3 h with a degradation rate of 90%. Since no active single-degrader was isolated from consortium SAAS79, the authors speculated that the synergistic effect among the species in the bacterial consortium may contribute to the degradation process [83]. Similarly, TADC7, a high performance thermophilic microbial consortium constructed by Wang et al. (2017) exhibited a stable and efficacious ability to degrade high doses of AFB1 (up to 5000 μg L^−1^) under extreme heat condition. Isolates from the consortium were found to be able to reduce AFB1 to varying degrees, although none was comparable to the efficacy of microbial consortium TADC7, indicating that TADC7 benefits from the synergistic effects [84].

During complex processing and storage, food and feed are probably co-contaminated by multiple, leading to more serious toxic effects than individual mycotoxin. Therefore, it is necessary to achieve effective co-degradation of multiple mycotoxins synchronously [85]. For instance, the co-existence of AFB1 and ZEA in agricultural products may have a higher hepatotoxicity than either of them alone [86]. TADC7, the aforementioned AFB1-degrading microbial community, was domesticated by co-culturing with AFB1 and ZEA, yielding the derived microbial consortium TMDC with a stable microbial composition and the ability to simultaneously degrade AFB1 and ZEA [87]. A promising new approach is the combination of compound probiotics (CP) with mycotoxin-degradation enzymes for the synchronous detoxification of AFB1 and ZEA. Compared to individual *Bacillus subtilis*, the combination with *Lactobacillus casein* and *Candida utilis* (1:1:1) significantly improved the degradability with a degradation rate of 45.49% and 44.90% for AFB1 and ZEA, respectively. The synergistic use of compound probiotics with mycotoxin-degradation enzymes (MDEs) from *Aspergillus oryzae* at a ratio of 3:2 could further improve degradation rates (63.95% and 73.51%) [9]. To reduce or eliminate the hazards of AFB1 and ZEA in broiler growth, CP of *Bacillus subtilis*, *Lactobacillus casei* and *Candida utilis* (7, 6 and 7 log CFU g^−1^) was added in broiler diets for a simultaneous biodegradation of the toxins. As a result, the CP addition decreased mycotoxin residues through positively regulating bacterial abundances of gut microbiota [88]. Moreover, the cell-free supernatant of compound probiotics (CFSCP) combined with MDEs has been demonstrated to be capable of alleviating the cytotoxic effects of AFB1 and ZEA on swine jejunal epithelial cells [89]. These findings shed light on the potential application of microbial consortia in the biodegradation of mycotoxins.

## 5. Mycotoxin Degradation by Recombinant Enzymes

Various microbiological techniques have emerged for eliminating or controlling mycotoxin over the past decades. Among them, on the investigation of enzymes that can transform mycotoxins to less toxic or nontoxic products have has been a prevalent topic [90]. A wide range of enzymes have been identified for mycotoxin degradation, including laccase, manganese peroxidase (MnP) and oxidase [91]. Although the identification, characterization and purification of mycotoxin-degrading enzymes (MDE) can sometimes be time consuming and fails to be cost-effective, the use of pure enzymes offers an attractive and unavoidable alternative to the application of whole bacterial cells, especially under conditions that are not conducive to the survival of microorganisms. Moreover, compared to the use of living microorganisms, enzymatic degradation has several superiorities such as ease-of-handling, reproducible and homogeneous performances, high efficiency and high specificity of action [23]. However, a number of MDE are naturally produced and stored in fungi intracellularly, making them particularly difficult to lysate and extract. Although various natural MDE were purified from microorganisms in recent years as reviewed in the previous literature [1,12,17,25], their application in real food and feed with complex matrices is very limited [90].

Mycotoxin degradation by a recombinant enzyme is particularly attractive because the clone and heterologous expression of enzymes via the genetic engineering technique allow for efficient production at lower economic and labor costs. For example, it is very challenging to obtain natural laccase from the fungi *Streptomyces* *cyaneus* CECT 3335 due to its slow growth rate and narrow optimum pH range. While the large-scale production of laccase was realized by the heterologous expression in *Escherichia coli* with the yield up to 104 mgL^−1^ [92]. Some studies concerning mycotoxin degradation by recombinant enzymes expressed in engineered strains are shown in Table 3. Yu et al. (2012) cloned and expressed a gene of peroxiredoxin (Prx) from *Acinetobacter* sp. SM04 in *E. coli*. The recombinant Prx was able to degrade ZEA in the presence of H_2_O_2_ efficiently. Moreover, nearly 90% of ZEA was degraded when this recombinant Prx was employed for the treatment of ZEA-contaminated corn [93]. However, the undesirable formation of inclusion bodies may limit the high overexpression of Prx. in *E. coli*. To eliminate the disadvantages, Tang et al. (2013) successfully expressed Prx as a secreted product in the eukaryote *S. cerevisiae*, which avoided the formation of inclusion bodies and complex purification step. Furthermore, S. cerevisiae conforms to the standards of GRAS by FDA in USA, which is particularly beneficial for the practical application of recombinant Prx in real matrices of foods and feeds [94]. Researchers have also tried to express the ZEA-degrading enzyme in *Pichia pastoris*, a robust fermentation organism with the ability to secrete protein into the medium. Xiang et al.(2016) reported a high level expressed lactone hydrolase ZHD with the specific activity of 4976.5 U mg^−1^ in the recombinant *P. pastoris* X3c derived from *Gliocladium roseum* by codon optimization and bio-brick method [95]. Bi et al. employed *P. pastoris* as a secretory expression system to express a zearalenone lactonase gene from *Neurospora crassa* (ZENC). The maximal enzyme activity of ZENC reached 290.6 U mL^−1^ in a 30-L fermenter using high-density fermentation. Moreover, ZENC was also found to be efficient in ZEN-containing materials with high degradation rate, providing a foundation for industrial application [96]. In 2018, Wang et al. expressed a novel lactonohydrolase Zhd518 in *E. coli* with high degrading activities against ZEA and its derivatives. The optimal temperature and pH of Zhd518 was 40 °C and 8.0, indicating that this enzyme can be used as an excellent candidate for ZEA detoxification in the food and feed industry [97]. Yang et al. (2017) reported the first successful expression of a ZEA-degrading enzyme ZHD101 in a probiotic strain *Lactobacillus reuteri* Pg4 without losing their probiotic properties. It is suggested that *L. reuteri* pNZ-zhd101 can be used as a probiotic feed additive with the function of ZEA-degradation [98].

Recombinant enzymes have also been used for the degradation of FB1. The process involves at least two enzymatic reactions, namely, de-esterification by a carboxylesterase resulting in hydrolyzed FB1 followed by an oxidative deamination of the hydrolysis product. Based on this, Heinl et al. (2010) isolated and heterologously expressed a carboxylesterase and an aminotransferase gene from *Sphingopyxis* sp. MTA144 for consecutive FB1 detoxification [99]. It is worth noting that the aminotransferase (FumI) is independent of oxygen, which is especially beneficial for its applications under oxygen limited conditions such as ensilaged forage and animal intestinal tract. FumI is tolerant to a wide range of temperature (6–50 °C) and pH (6–10),with the optimum activity at 35 °C and pH 8.5 [100]. In 2014, the cloning, purification and characterization of two amidases from *A. niger* was reported by Dobritzsch et al. these thermo stable amidases were demonstrated to possess OTA-hydrolyzing activity with optimal activity at pH 6 and 66 °C [101]. In a recent study, Zhang et al. purified and identified a novel OTA degrading enzyme, N-acyl-L-amino acid amidohydrolase (*Af*OTase) from *A. faecalis* DSM and expressed it in *E. coli. Af*OTase exhibited commendable thermal and pH stability [102]. Carere et al. identified a dehydrogenase with the capability to transform DON to the less toxic 3-keto-DON [103]. Cytochrome P450s play important roles in the metabolism of endogenous and exogenous substrates. Rawal et al. (2010) described the heterologous expression and functional characterization of P4503A37 from a turkey liver with high aflatoxin B1 epoxidation activity [104]. Ito et al. (2013) reported a cytochrome P450 system with DON-catabolic activity in *Sphingomonas* sp. strain KSM1. This enzyme system can transform DON to 16-hydroxy-deoxynivalenol. Similar degrading abilities were also demonstrated toward nivalenol and 3-acetyl deoxynivalenol, toxins that frequently co-occur with DON [105]. These findings provide a biochemical and genetic basis for economic production of recombinant enzymes to eliminate the toxic threat.

To manage the co-contamination of different mycotoxins, the recombinant genetic engineering of two or more enzymes for the degradation of multiple mycotoxins has become a new development direction. Azam et al. (2019) reported a novel bifunctional recombinant fusion enzyme (ZHDCP) combining zearalenone hydrolase (ZHD) and carboxypeptidase (CP) by genetic engineering technology. The bifunctional enzyme was able to completely degrade ZEA and OTA in 2 h and 30 min, respectively. The final products were less harmful with reduced cell death and mitochondrial apoptosis. The result demonstrated that bifunctional purified enzyme can be more advanced and superior than the single strain or enzyme [106]. Another feasible route would be the use of a single mycotoxin-degrading enzyme with wide substrate specificity on multiple mycotoxins for the treatment of two or even more mycotoxins co-contamination. A recent study revealed that eight recombinantly produced manganese peroxidase (MnP), responsible for lignin oxidative depolymerization in fungi, could degrade four major mycotoxins including AFB1, ZEA, DON and FB1 in the presence of a dicarboxylic acid malonate [107].

**Figure 2:**
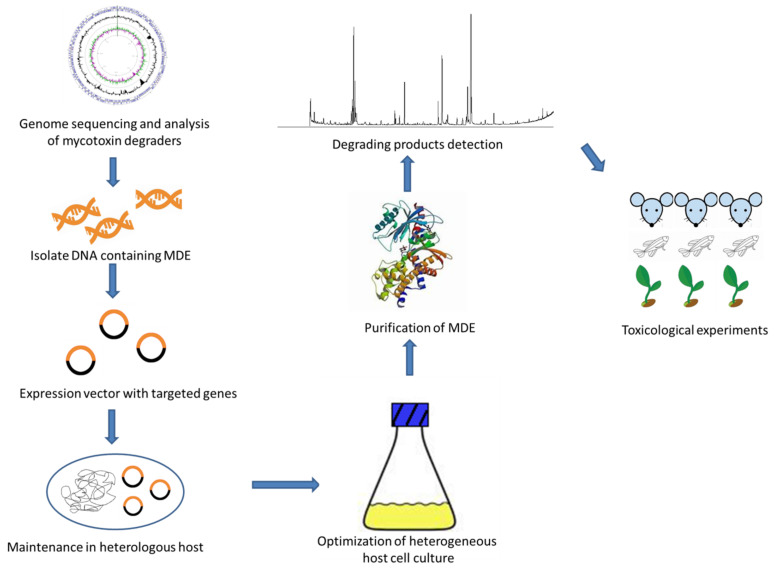
The mechanism of heterogeneous expression of mycotoxin degradation enzyme (MDE).

The above studies made contributions to the application of genetic engineering methods to mycotoxins degradation; however, the commercial application of recombinant MDE is still far from achievable. To provide a foundation for the industrial application of recombinant MDE, here we suggest a research route as shown in Figure 2. The screening and cloning of new degradation pathways and MDE genes are of great importance to the development of the entire industry. Novel metagenomics screening surveys may serve as the resources for the acquisition of diverse MDE from unexplored complex natural samples. To realize the efficient production of MDE, optimization of incubation and purification conditions including temperature and pH is necessary. Moreover, before the applying MDEs in real matrices, the possible toxic effects of the degrading products should be fully evaluated.

## 6. Application, Prospect and Challenges

### 6.1. Unexplored Mechanisms of Mycotoxins Degradation

Over the last decade, knowledge about new organisms and metabolic pathways on the biological mycotoxin degradation has grown rapidly. Tracking the fate of converted products from mycotoxins created through the metabolism of living organisms or via enzyme reaction is necessary for commercial application. However, most studies report on preliminary investigation of degradation ability without characterizing the intermediate metabolites and final products that are sometimes hardly analyzable under laboratory conditions. A full understanding of the structure and stability of degradation products could provide useful information for evaluating their potential side-effects and health impact. Traditional analysis methods, based on instruments such as LC-MS/MS, GC-MS/MS, and nuclear magnetic resonance (NMR) were not able to detect all the metabolites involved in the conversion of mycotoxins. New strategies for elucidating a pathway of biodegradation must be developed. Wang et al. (2020) reported a metabolomics-guided analysis to reveal metabolism during DON degradation catalyzed by bacterial consortium IFSN-C1 [108]. In the study of Pinedo et al. (2018) isotopic labeling experiments with ^13^C-labeled substrates helped to reveal the detoxification pathway of patulin to desoxypatulinic acid (DPA) by *Rhodotorula kratochvilovae* LS11 via the hydrolysis of the γ-lactone ring and, subsequently, enzymatic modifications [109]. With the development of genome sequencing technology and gene bank database, advanced high-throughput approaches, such as functional metagenomic and comparative transcriptomic, provide robust tools for the identification of novel genes responsible involved in biodegradation. In the study of Yu et al. (2019), community-level BPA degradation pathway was constructed based on the integrated data of metabolites and metagenomics [110]. Moreover, Zhang et al. (2019) performed a transcriptome analysis to investigate mechanisms involved in OTA degradation by *Yarrowia lipolytica* Y-2. Notably, the gene ECM14 encoding putative metallocarboxypeptidase ECM14 was up-regulated in *Y. lipolytica* Y-2 when treated with OTA [111]. As supplementary of experimental research, computational study with bioinformatic analysis could help to improve the understanding of the enzyme functions. Tomin et al. (2019) performed an extensive computational study to investigate the affinity of an aflatoxin oxidase (AFO) from *Armillariella tabescens* toward AFB1, and elucidated the details of the enzyme structure. The results of this study strongly support that AFO is a typical member of the dipeptidyl peptidase III family [112].

### 6.2. Biosafety Evaluation of Degradation Products

Over recent years, mycotoxin degradation by biological methods has been studied extensively. However, the possible toxicity of residual by-products formed in the degradation process has been overlooked. It is noticeable that toxins are not necessarily degraded into water and carbon dioxide [113]. It is therefore important to evaluate the biosafety of the degradation products to explain the mechanism and of biological mycotoxin degradation and lay a foundation for its commercial application. So far, only a limited number of reports have elucidated the structure of some converted products and performed short-term preliminary tests for the estimation of their toxicity. Zhu et al. (2017) summarized the models and methodologies for toxicity determination of mycotoxins and by-products in a review paper [11]. Hosts that are used in toxicity tests vary from animals that are ranked highly within the evolutionary tree to bacteria. Specific cell lines, such as MCF-7 cells, may be more suitable for testing certain toxins including estrogenic activity and cytotoxicity. Recently, the zebrafish has emerged as an important toxicological model, based on which easier routes have been developed for the evaluation of teratogenicity [114]. Haq et al. (2016) employed the zebrafish embryo toxicity assay (ZETA) to evaluate the teratogenicity of OTA and its degradation product OTα. Results of this study suggested that ZETA could be regarded as a useful and sensitive tool for evaluating the safety of detoxification strategies for contaminants including microbial toxins [115]. Since rigid parameters for the assessment of the safety of detoxification products have not been yet sufficiently established, it is necessary to have a full understanding of any associated risks before industrial applications of biological degradation agents.

### 6.3. Limited Practical Application in Real Matrices

Despite the extensive research on the biological degradation of mycotoxins by microorganisms and their enzymes, the practical application in food and feeds matrices is limited. Since the degradation process under conditions of commercial-scale production is much more complex, laboratory experiments might not always reflect practices in industrial processing. The possible reasons are that the degradation activities can be easily affected by multiple factors, such as co-factor requirement, pH, ionic-strength, temperature, etc. Given the above situation, novel non-pathogenic strains with high toxin-degradation performance and good stability are in considerable demand for practical use. Besides, the survival and adaptability of microorganisms under simulated conditions should be fully evaluated for mycotoxin detoxification in animal guts. Moreover, from an economical point of view, the cost of a lengthy incubation time and the fermentation materials should be lowered. For example, studies reported in the literature have found fermentation with LAB was an effective approach for mycotoxin degradation as summarized in an excellent review of Adebiyi et al. (2019) [116]. This food-processing technique not only provides a cheap, readily available and sustainable strategy for mycotoxin control, but also ensure the beneficial sensorial, nutritional and health composition of food. LAB strains with antifungal activity are also be reported to contribute to the safety of malt modification. In a pilot-scale malting trial with barley grains infected with *F. culmorum*, the application of *Lactobacillus reuteri* R29 cfs produced in 3 °P substrate successfully reduced mycotoxin DON by 83%, which represents a practical and inexpensive alternative to mycotoxin control in the malt industry [117].

## 7. Conclusions

The widespread contamination of mycotoxins has attracted worldwide attention. Many strategies have been developed for mycotoxin removal, and biological degradation is considered as the safest strategy. Probiotics, which possess plenty of beneficial properties, are reported to be useful for mycotoxins removal. The application of a microbial consortium provides a promising solution for the effective co-degradation of multiple mycotoxins. Besides, with the development of genetic engineering technology, recombinant degrading enzymes are increasingly favored in biological detoxification. It is necessary to develop recombinant degrading enzymes capable of degrading multi-toxins. Currently, however, most biological detoxification technologies have not yet been developed for the commercial scale, since a thorough understanding of the degradation pathways and the potential health effects of the degradation products does not yet exist. To facilitate the commercial application of these approaches, future studies should focus on explanining of the detailed degradation mechanisms, conducting-toxicology studies of metabolites, and developing novel analytical methods. Given the great diversity of complex matrices, further research is recommended to highlight the field-applicable degradation technologies for the control of mycotoxins. In the future, more efforts should be dedicated to exploring strategies for mycotoxins biodegradation that are cost-effective, highly efficient, instrumently simple and easy to operate for large-scale application in industry.

## Figures and Tables

**Table 1:** Chemical structure, toxic groups, biological effects of main mycotoxins and producing fungi.

Mycotoxins	Chemical Structure	Main Toxic Groups	Main Degradation Products	Organ/System Affected	Main Clinical Signs	Producing Fungi
Aflatoxins (B1, B2, G1, G2)	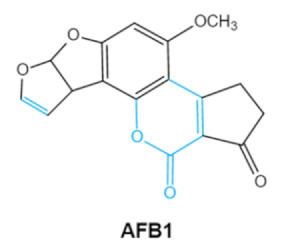 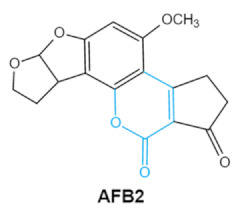 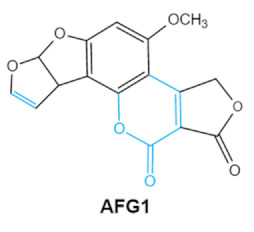 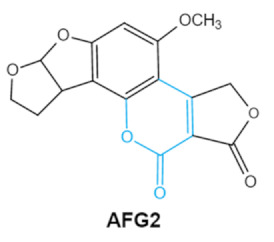	Lactone ring Double bond in difuran ring moiety	AFB1-8,9dihydrodiol, AFB1-8,9-epoxide, dihydrohydroxyaflatoxin B1,	Liver, kidney, immune system	Hepatitis, carcinogenic, abdominal pain, vomiting, increased susceptibility to disease, immunosuppressive and carcinogenic effects	*Aspergillus. Flavus* *A. Parasiticus* *A. nomius*
Zearalenones (ZEA)	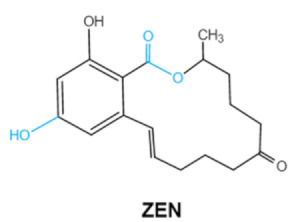	Lactone ring C-4 hydroxyl group	α-/β-zearalenol, α-/β-zearalanol,zearalenone-4-sulfate, 1-(3,5-dihydroxyphenyl)-6′-hydroxy-l’-undecen-l0′-one, (5S)-5-({2,4-dihydroxy-6-[(1E)-5-hydroxypent-1-en-1-yl]benzoyl}oxy)hexanoic acid	Reproductive tract, mainly female	Hyperestrogenism, Reproductive disorders	*Fusarium graminearum* *(F. roseum)* *F. culmorum* *F. equiseti* *F. cerealis* *F. verticillioides* *F. incarnatum*
Ochratoxins (A,B,C) (OTs)	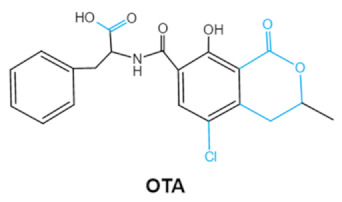 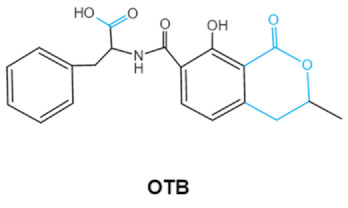 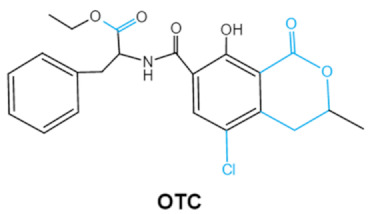	Isocoumarin moiety Carboxyl group of the phenylalanine moietyCl group	L-βphenylalanine, OTα	Liver, kidney, immune system, inhibit RNA, DNA and protein synthesis in kidney	Nephritis, enlargement of kidney and hepatitis	*A. ochraceus* *A. carbonarius* *A. niger* *P. verrucosum* *P. nordicum*
Fumonisins FBs (B1, B2)	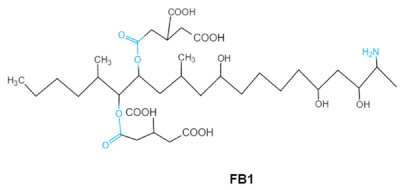 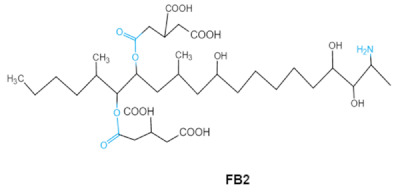	Two tricarballylic acid side chains Free amino group	2-oxo-12,16-dimethyl-3,5,10,14,15-icosanepentol hemiketal, NacetylAP1,	Lungs and heart (pig), central nervous system (horse), liver, immune system	Porcine pulmonary edema (PPE), equine leukoencephalomalacia	*Fusarium* section *Liseola*
Trichothecenes (DON, T-2, HT-2) TCNs	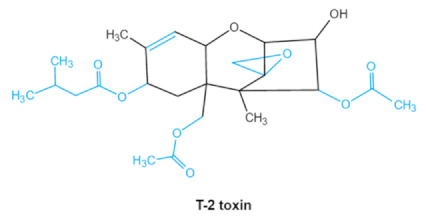 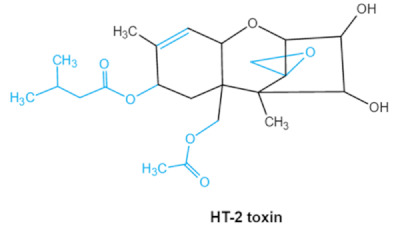 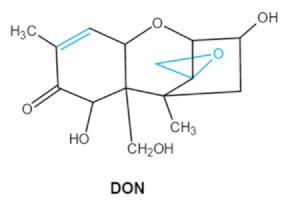	Epoxide group Acylated side groups C9-10 double bond	HT-2 toxin, T-2 triol, T-2 tetraol, de-epoxy T-2 tetraol, 3α,7α,15α-triacetoxy-deoxynivalenol, de-epoxy deoxynivalenol, 3-acetyldeoxynivalenol, diaacetoxydeoxynivalenol. Epoxymonoacetoxyscirpenol, de-epoxyscirpentrio	Central nervous system, gastrointestinal tract, liver, immune system	Anorexia, vomiting, abdominal pains, cardiovascular dysfunction	*F. acuminatum* *F. sporotrichioides* *F. langsethiae* *Fusariumgraminearum*, *F. culmorum* *F. cerealis* *F. culmorum* *F. graminearum* *F. sporotrichioides* *F. poae*
Patulin PAT	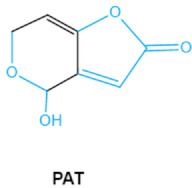	Furan, pyran or lactone ring Hemiacetal	Ascladiol, hydroascladiol, desoxypatulinic acid, 3-keto-5-hydroxypentanal, glyoxylic acid	Gut epithelium, liver, kidney, immune system	Oral and epithelial lesion, loss of appetite Nausea, vomitin, gastric ulcers	*Penicillim expansum Bysochlamis nívea* *Aspergillus clavatus* *P. griseofulvum*

**Table 2:** Degradation of mycotoxins by probiotic bacterial strains reported in the last decade.

Mycotoxins	Microorganism	Reduction Rate (%)	Toxin Level	Degradation Condition	Reference
AFB1	*Streptomyces cacaoi subsp. Asoensis* K234	88.34 ± 15.62	1 μg mL^−1^	5 days, 28 °C,170 rpm liquid LB medium	Harkai et al. (2016)
	*Streptomyces luteogriseus* K144	79.93	1 μg mL^−1^	5 days, 28 °C,170 rpm liquid LB medium	
	*Bacillus licheniformis* CFR1	94.73 ± 1.09	500 ppb	liquid nutrient broth (NB) at 37 °C, 72 h	Rao et al. (2017)
T-2	*P. pentosaceus* KTU05-10	78.0	12.8–19.5 μg kg^−1^	malting wheat grains with bacterial suspension at 18 °C for 30 min	Juodeikiene et al. (2018)
	*Saccharomyces pastorianus* A15	31.0	5000 µg L^−1^	15 °C, 120 rpm, four days 11.5° Plato wort	Nathanail et al. (2016)
HT-2	*P. pentosaceus* KTU05-10	79.0	258–819 μg L^−1^	malting wheat grains with bacterial suspension at 18 °C for 30 min	Juodeikiene et al. (2018)
ZEA	*P. acidilactici*	38.0	19.5–873.7 μg L^−1^	malting wheat grains with bacterial suspension at 18 °C for 30 min	Juodeikiene et al. (2018)
	*Streptomyces rimosus* (K145, K189)	100.0	1 μg mL^−1^	5 days, 28 °C,170 rpm liquid LB medium	Harkai et al. (2016)
DON	*P. pentosaceus* KTU05-10	47.0	3370–6930 μg kg^−1^	malting wheat grains with bacterial suspension at 18 °C for 30 min	Juodeikiene et al. (2018)
	*Saccharomyces pastorianus* A15	15.0	400 µg L^−1^	11.5° Plato wort, 15 °C, 120 rpm for 4 days	Nathanail et al. (2016)

**Table 3:** Degradation of mycotoxin by recombinant enzymes.

Enzyme	Gene Source	Target Toxin	Degrading Products	Expression System	Degrading Properties	Degradation Mechanism	Reference
Cytochrome P450 3A37	Turkey liver	AFB1	*exo*-AFBO aflatoxin Q1	*E. coli*	*exo*-AFBO, K_m_: 287 ±21 µmol L^−1^ V_max_: 1.45 ± 0.07 nmol min^−1^ P450 aflatoxin Q1, K_m_: 302 ±51 µmol L^−1^ V_max_: 7.86 ±0.75 nmol min^−1^	Epoxidation	Rawal et al. (2010)
Peroxiredoxin (Prx)	*Acinetobacter* sp. SM04	ZEA	NM	*E. coli*	The optimum degradation pH and temperature was 9.0 and 70 °C in presence of H_2_O_2_	Oxidation	Yu et al. (2012)
Lactonohydrolase Zhd518	*Clonostachys rosea*	ZEA	NM	*E. coli*	Activity of 207.0 U mg^−1^ with the optimal temperature and pH at 40 °C and 8.0	NM	Wang et al. (2018)
Peroxiredoxin	*Acinetobacter* sp. SM04	ZEA	NM	*Saccharomyces cerevisiae*	Optimal activity at pH 9.0, 80 °C and H_2_O_2_ concentration of 20 mmol L^−1^ Thermal stable, alkali resistance	Oxidation	Tang et al. (2013)
Lactone hydrolase ZHD	*Gliocladium roseum*	ZEA	α-zearalenol and β-zearalenol	*Pichia pastoris*	Enzyme activity in shake flask fermentation was 22.5 U mL^−1^ with the specific activity of 4976.5 U mg^−1^ The maximum enzyme activity of the supernatant was 150.1 U mL^−1^ in 5-L fermenter	Cleavage of lactone ring	Xiang et al. (2016)
Lactonohydrolase	*Clonostachys rosea*	ZEA	1-(3,5-dihydroxy-phenyl)-10-hydroxy-1-undecen6-one	*Lactobacillus reuteri Pg4*	Did not affect cell growth, acid and bile salt tolerance	Cleavage of lactone ring	Yang et al. (2017)
Lactonase	*Neurospora crassa*	ZEA	NM	*Pichia pastoris*	Optimal activity at pH 8.0 and 45 °C, highly stable at pH 6.0–8.0 for 1 h at 37 °C, the maximal enzyme activity reached 290.6 U mL^−1^ in 30-L fermenter	NM	Bi et al. (2018)
Carboxylesterases, type B	*Sphingopyxis* sp. MTA144	FB1	NM	*E. coli*	NM	Deesterification	Heinl et al. (2010)
Aminotransferases, class III		HFB1			Oxygen independence, temperature range 6–50 °C with an optimum at 35 °C, and pH adaptation 6–10 with an optimum at pH 8.5	Deamination	
Aminotransferase FumI	*Sphingopyxis* sp. MTA144	HFB1	NM	*E. coli*	Optimal activity at pH 8.5 and 35 °C, low salt concentration, the kinetic parameters K_m_ = 1.1 μmol L^−1^ and k_cat_ = 104 min^−1^	Eamination	Hartinger et al. (2011)
Putative amidase	*Aspergillus niger*	OTA	NM	NM	Thermostable, optimal activity at pH 6.0 and 66 °C	Hydrolysis	Dobritzsch et al. (2014)
N-acyl-L-amino acid amidohydrolase	*Alcaligenes faecalis*	OTA	β-phenylalanine	*E. coli*	Optimal activity at pH 6.5 and 50 °C	OTA amide bond hydrolysis	Zhang et al. (2019)
Dehydrogenase DepA	*Devosia mutans* 17-2-E-8	DON	3-keto-DON	*E. coli*	NM	Oxidation of C3 position	Carere et al. (2017)
Cytochrome P450	*Sphingomonas* sp. strain KSM1	DON	16-hydroxy-deoxynivalenol	*E. coli*	k_cat_/K_m_ of 6.4 mmol L^−1^ s^−1^	Hydroxylation	Ito et al. (2013)
Fusion ZHDCP enzyme	*Clonostachy rosea* *B. amyloliquefaciens* ASAG	ZEA OTA	HZEA, DZEA, OTAα	*E. coli*	100% degradation rate at pH 7 and 30 °C in 2 h	Hydrolysis Removal of an amino acid from the end of a peptide chain	Azam et al. (2019)
Manganese peroxidase	*Irpex lacteus*, *Phanerochaete chrysosporium*, *Ceriporiopsis subvermispora*, *Nematoloma frowardii*	AFB1 ZEA DON FB1	AFB1-8,9-epoxide	*E. coli*	In the presence of dicarboxylic acid malonate	Oxidoreduction	Wang et al. (2019)

NM, not mentioned.
